# Evaluation of lifestyle behaviors, anxiety and depression in patients with hematologic disorders

**DOI:** 10.1097/MD.0000000000035863

**Published:** 2023-11-17

**Authors:** Zhexiang Kuang, Bin Zhang, Xia Li, Jingyu Zhao, Jing Xu, Zhiqiong Wei, Liyun Li, Jin Dong, Xiao Yu, Juan Li, Juanjuan Zhao, Baoxin Shi

**Affiliations:** a Hospice Research Center of Tianjin Medical University, Tianjin, China; b State Key Laboratory of Experimental Hematology, National Clinical Research Center for Blood Diseases, Haihe Laboratory of Cell Ecosystem, Institute of Hematology and Blood Diseases Hospital, Chinese Academy of Medical Sciences and Peking Union Medical College, Tianjin, China; c Tianjin Institutes of Health Science, Tianjin, China; d Department of Mathematics and Statistics, La Trobe University, Melbourne, Australia.

**Keywords:** anxiety, association, depression, hematologic disorders, latent class analysis, lifestyle behaviors

## Abstract

Patients with hematologic disorders may experience anxiety and depression due to their immunocompromised status and potential side effects of therapies. Healthy lifestyle behaviors might enhance the mental health. To evaluate the association of both separate and clustering pattern lifestyle behaviors with anxiety and depression in hematological patients, healthcare providers can develop future initiatives that respond to the specific needs of this population. A total of 185 patients with hematologic disorders were enrolled in this cross-sectional study. Linear regression analysis was performed to measure the association of separate lifestyles with anxiety and depression. Latent class analysis was further conducted to identify homogeneous and mutually exclusive lifestyle classes, and the logistic regression was then used to assess the relationship between class memberships and symptoms of anxiety and depression. The study found sleep quality was correlated with anxiety and depression. Nevertheless, no association of anxious and depressive symptoms with sitting and exercise, dietary habits, toxicant exposure, drinking, and smoking, in either the overall patient population or patients classified by hematologic neoplasms. Two latent classes of lifestyle behaviors were further identified, but the class memberships were independent of anxiety and depression. The study suggested that promoting sleep quality was a viable intervention for patients with hematologic disorders. However, the clustering pattern of lifestyles may not be a reliable indicator of psychological issues.

## 1. Introduction

Hematologic disorders are a variety of disorders including rare congenital diseases, anemia, bleeding diseases, and hematologic neoplasms such as lymphoma, myeloma and leukemia. Prior studies indicated that individuals with hematologic disorders were susceptible to psychological issues because of their weakened immune system and potential side effects of treatments.^[1–4]^ Anxiety and depression are common mental health concerns among patients.^[5]^ These negative emotions are detrimental to their motivation and compliance and therefore impair the disease outcomes.^[6,7]^ Although anxiety and depression are affected by the disease burden, they are also influenced by lifestyle behaviors that can be modified to improve the health status.^[8–10]^ For instance, consuming a healthy diet may reduce the risk of infections for hematological patients whose immune systems are weakened, and thus enhance their quality of life which is positively associated with mental health.^[11,12]^

The relationship between lifestyles and mental conditions has been an increasing concern among healthcare providers. Previous studies suggested that multiple lifestyles are significantly associated with anxiety and depression.^[13–15]^ However, few studies focused on the synergistic effect of lifestyles on mental health. Lifestyle behaviors are clustered and are unlikely to occur by chance.^[16]^ For instance, people exposed to physical or chemical substances in the workforce are prone to sleep disturbance.^[17]^ Moreover, tobacco exposure may reinforce alcohol dependence, poor diet and physical inactivity.^[18–20]^ Hence, it is necessary to identify both the individual and clustering pattern of lifestyle behaviors for patients to promote mental well-being.

To the best of our knowledge, no study has been conducted to investigate the association of separate and combined health behaviors with anxiety and depression among patients with hematologic disorders. In this study, we initially evaluated the anxious and depressive symptoms and the lifestyle behaviors of patients classified by hematologic neoplasms. Simple and multiple linear regression were performed to measure the association of separate lifestyles with anxiety and depression. We further examined the clustering of multiple lifestyles to identify risk factors for anxiety and depression by the latent class model. The aim of this study was to explore the associations among sitting and exercise, dietary habits, sleep quality, toxicant exposure, drinking and smoking with anxiety and depression in patients with hematologic disorders, and to provide adequate nursing interventions for these patients to achieve better health outcomes.

## 2. Methods

### 2.1. Study design and sample

We recruited 185 patients from February 2020 to December 2021 Institute of Hematology and Blood Diseases Hospital (IHBDH), which is the largest national research-oriented medical institution for blood diseases in China. Patients met the following criteria were included in this study: Patients over age 18 years, and had a diagnosis of hematologic disorders, and received the immunosuppressive therapy, targeted therapy or chemotherapy, and agreed to participate in this research after providing verbal informed consent. Patients were excluded if they had any mental illness historically or currently.

The anxious and depressive symptoms of each patient were collected by the clinical research nurse through a 40-minute confidential and intimate conversation before receiving the treatment. Meanwhile, the information of lifestyle patterns was obtained by patients completing the online questionnaires. The demographic and clinical characteristics can be extracted from electronic medical records. All these data were documented electronically by the statistician. This cross-sectional study was approved by the Ethics Committee of IHBDH (DC2020003-EC-1).

### 2.2. Measures

#### 2.2.1. Sitting and exercise.

Sitting was defined as the time spending per day in motorized transport, and learning, reading, using technologies (watching televisions, using mobiles and laptops) at work, at home and in leisure time. It was classified into low sitting time (<8 hours per day) and long sitting time (≥8 hours per day) according to the previous study.^[21]^ Exercise was defined as weekly minutes of moderate aerobic exercise (social dancing, swimming, running and brisk walking), and it was categorized into sufficient (≥150 minutes) and insufficient (<150 minutes) based on the prior research.^[22]^

#### 2.2.2. Dietary habits.

Dietary habits focused on whether patients maintain regular meals, and the weekly frequency of the takeout food consumption and fruit intake. The regular mealtime was a dichotomous variable, and it was defined as eating meals at regular intervals throughout the day. For both takeout food consumption and fruit intake, no more than 1 day per week was regarded as low frequency, otherwise, it was regarded as medium-high frequency.

#### 2.2.3. Sleep quality.

The Pittsburgh Sleep Quality Index (PSQI) was used to measure patients’ quality of sleep in recent 1 month. The PSQI questionnaire contains 19 self-reported items, yielding 7 components (subjective sleep quality, sleep latency, sleep duration, habitual sleep efficiency, sleep disturbances, use of sleeping medication and daytime dysfunction) that generate a global score. A higher score indicates a lower poor sleep. A global score of no more than 5 indicates good sleep, otherwise, it is considered poor sleep. The Chinese version of the PSQI was translated by Liu^[23]^ in 1996. It has been subsequently applicated in different populations, including those with blood disorders,^[24–28]^ and all indicate the Chinese version is a reliable and valid instrument for assessing sleep quality. In this study, the PSQI showed a fair Cronbach’s alpha value of 0.693.^[29]^

#### 2.2.4. Toxicant exposure.

Toxicant exposure refers to contact with hazardous substances in the workplace, including both physical and chemical hazards such as carcinogenicity and corrosion. Patients with at least 1 year of toxicant exposure were regarded as exposed population.

#### 2.2.5. Drinking and smoking.

Drinking alcohol was evaluated by the number of standard drinks each week. A Chinese standard drink is any drink containing 10 grams of pure ethanol.^[30]^ nondrinkers were individuals who never drank or consumed less than 1 standard drink, and drinkers were classified as those consuming at least 1 standard drink each week in the last year. Smoking was classified into smokers and nonsmokers. Participants with electronic or conventional cigarette use over 1 year in current or history were considered as smokers.

#### 2.2.6. Anxiety.

The severity of anxiety symptoms was measured using the Hamilton Anxiety Rating Scale (HAM-A). Both somatic anxiety and psychic anxiety were assessed based on the 14 items. Each item is scored from 0 to 4, indicating symptoms from not present to severe. This calculation generates a total score in the range of 0 to 56, where ≤ 17 indicates mild severity, 18 to 24 mild to moderate severity, and 25 to 30 moderate to severe.^[31]^ The Chinese version of the HAM-A is a valid and reliable instrument,^[32]^ and it had a fair Cronbach’s alpha 0.685 in this study.^[33]^

#### 2.2.7. Depression.

Hamilton Depression Rating Scale (HAM-D) consists of 17 items designed to assess the severity of a patient’s depression. Each item is scored independently based on a 3 or 5-point Likert-type scale. A higher score indicates more serve depressive symptoms. The sum of the responses ≤ 7 indicates not depressed, 8 to 13 mild, 14 to 18 moderate, 19 to 22 severe, >23 very severe.^[34]^ The Chinese version of HAM-D has a satisfactory reliability,^[35]^ with a Cronbach’s alpha value of 0.712 in this study, indicating an acceptable internal consistency.^[36]^

#### 2.2.8. Sociodemographic characteristics.

Demographic information consists of age, sex, body mass index (calculated as weight in kilograms divided by height in meters squared), education background, employment, and type of health insurance, most of which are categorical variables except that age is a continuous one. According to the World Health Organization, body mass index was categorized into underweight (≤ 18.5 kg/m^2^), normal (18.5 - 24.9 kg/m^2^) and overweight/obese (≥ 25.0 kg/m^2^). The height and weight were measured by nurses once patients were admitted to IHBDH. Some hematologic disorders can be categorized as malignant depending on the World Health Organization classification.^[37]^

### 2.3. Statistical analysis

Simple and multiple linear regression were performed to measure the association of lifestyles with anxiety and depression. The *t* test or *Wilcoxon rank-sum* test was performed to compare 2 sets of numerical data. The *Pearson’s chi-square* test or *Wilcoxon rank-sum* test was used to compare the counting data and grade data. The Spearman correlation coefficient was conducted to assess the correlation of anxiety and depression with sleep quality after the normality test.

Latent class analysis was conducted to discover homogeneous, mutually exclusive lifestyle classes, depending on responses to 9 indicators of 5 lifestyle behaviors outlined above. The optimal number of latent classes was determined by conducting a series of latent class analysis specifying 2 to 5 classes. The lower values of Akaike information criterion statistics, the Bayesian information criterion, and the sample-size-adjusted Bayesian information criterion indicated a better model fit. Considering the sample size, sample-size-adjusted Bayesian information criterion was used to select the appropriate number of classes in this study. Logistic regression was then performed to examine the relationship between combined lifestyles and symptoms of anxiety and depression. R (version 4.0.2) was used for data process and statistical analysis.

## 3. Results

### 3.1. Descriptive characteristics of the sample

The study engaged a total of 185 patients aged 18 to 65 (mean = 42.10, SD = 13.12) years old, comprising 112 patients with hematologic neoplasms (male = 70) and 73 patients with non-hematologic neoplasms (male = 33). Among the whole patients, 3.78% (n = 7) were underweight, 51.89% (n = 96) had normal weight and 44.33% (n = 82) had obesity. The percentage of those whose education background were secondary school or less, high school or associate, and university were 41.08% (n = 76), 37.84% (n = 70), and 21.08% (n = 39). Concerning the health insurance, the proportions of subjects had Urban Employee Basic Medical Insurance and New Cooperative Medical Scheme were similar, with 44.33% and 43.78%, respectively. In addition, majority of the participants have occupations (n = 144, 77.84%).

### 3.2. Subgroup analysis of patients classified by hematologic neoplasms

Hematologic neoplasms included leukemia (n = 69, 61.61%), lymphoma (n = 1, 0.89%), myelodysplastic syndromes (n = 39, 34.82%), and myeloproliferative Neoplasms (n = 3, 2.68%). Patients with non-hematologic neoplasm were mainly diagnosed with aplastic anemia (n = 54, 73.97%), autoimmune hemolytic anemia (n = 13, 17.81%), and pure red cell aplasia (n = 6, 8.22%). Table 1 presents the difference of sociodemographic characteristics, lifestyle behaviors and mental conditions between the hematologic neoplasms and the non-hematologic neoplasms. Patients with hematologic neoplasms were older and more male than the non-hematologic neoplasms (*P* < .001 and *P* = .031). There were no differences of lifestyles, anxiety and depression in participants classified by hematologic neoplasms (*P* > .05).

**Table 1:** Characteristics of the study population.

	Total population n = 185	Hematologic neoplasms n = 112	Non-hematologic neoplasms n = 73	*P*
Sociodemographics				
Age (years, mean ± SD)	42.10 ± 13.12	45.05 ± 12.64	37.56 ± 12.60	<.001
Sex (N (%))				
Male	103 (55.68)	70 (62.50)	33 (45.21)	.031
Female	82 (44.32)	42 (37.50)	40 (54.79)	
Body mass index (kg/m^2^, N (%))				
Underweight	7 (3.78)	2 (1.79)	5 (6.85)	.452
Normal	96 (51.89)	59 (52.68)	37 (50.68)	
Overweight/obese	82 (44.33)	51 (45.53)	31 (42.47)	
Education (N (%))				
Secondary School or Less	76 (41.08)	51 (4554)	25 (34.25)	.127
High School/Associate	70 (37.84)	40 (3571)	30 (41.09)	
University	39 (21.08)	21 (1875)	18 (24.66)	
Health insurance (N (%))				
UEBMI	82 (44.33)	50 (44.64)	32 (43.84)	.980
URBMI	22 (11.89)	17 (15.18)	5 (6.85)	
NCMS	81 (43.78)	45 (40.18)	36 (49.31)	
Paid employment (N (%))				
Yes	144 (77.84)	92 (82.14)	52 (71.23)	.118
No	41 (22.16)	20 (17.86)	21 (28.77)	
HAM–A score (median, min–max)	8 (0–23)	8 (0–23)	8 (1–21)	.717
Levels of anxiety (N (%))				
Mild (Scores ≤ 17)	177 (95.68)	106 (94.64)	71 (97.26)	.483
Mild to moderate (Scores of 18–24)	8 (4.32)	6 (5.36)	2 (2.74)	
HAM–D score (median, min–max)	13 (0–30)	13 (0–30)	12 (3–30)	.273
Levels of depression (N (%))				
Not depressed (Scores ≤ 7)	31 (16.75)	16 (14.29)	15 (20.55)	.324
Mild (Scores of 8–13)	67 (36.22)	43 (38.39)	24 (32.88)	
Moderate (Scores of 14–18)	53 (28.65)	28 (25.00)	25 (34.24)	
Severe (Scores of 19–22)	21 (11.35)	16 (14.28)	5 (6.85)	
Very severe (Scores ≥ 23)	13 (7.03)	9 (8.04)	4 (5.48)	
Lifestyle behaviors				
Sitting time (N (%))				
<8 h per day (low)	126 (68.11)	82 (73.21)	44 (60.27)	.092
≥8 h per day (high)	59 (31.89)	30 (26.79)	29 (39.73)	
Exercise time (N (%))				
<150 min per week (insufficient exercise)	168 (90.81)	101 (90.18)	67 (91.78)	.914
≥150 min per week (sufficient exercise)	17 (9.19)	11 (9.82)	6 (8.22)	
Regular mealtime (N (%))				
Yes	127 (68.65)	79 (70.54)	48 (65.75)	.601
No	58 (31.35)	33 (29.46)	25 (34.25)	
Takeout food consumption (N (%))				
≤1 days per week (low frequency)	156 (84.32)	94 (83.93)	62 (84.93)	<.99
≥2 days per week (medium–high frequency)	29 (15.68)	18 (16.07)	11 (15.07)	
Fruit intake (N (%))				
≤1 days per week (low frequency)	18 (9.73)	12 (10.71)	6 (8.22)	.094
≥2 days per week (medium–high frequency)	167 (90.27)	100 (89.29)	67 (91.78)	
Sleep quality (N (%))				
Good	102 (55.14)	63 (56.25)	39 (53.42)	0.760
Poor	83 (44.86)	49 (43.75)	34 (46.58)	
Toxicant exposure (N (%))				
Unexposed	161 (87.03)	99 (88.39)	62 (84.93)	.645
Exposed	24 (12.97)	13 (11.61)	11 (15.07)	
Drinking (N (%))				
<1 standard drink per week	155 (83.78)	89 (79.46)	66 (9041)	.077
≥ 1 standard drink per week	30 (16.22)	23 (20.54)	7 (9.59)	
Smoking (N (%))				
Nonsmoker	121 (65.41)	68 (60.71)	53 (72.60)	.133
Smoker	64 (34.59)	44 (39.29)	20 (27.40)	

HAM-A = Hamilton Anxiety Rating Scale, HAM-D = Hamilton Depression Rating Scale, NCMS = New Cooperative Medical Scheme, UEBMI = Urban Employee Basic Medical Insurance.

### 3.3. Association of separate lifestyle behaviors with anxiety and depression

We conducted linear regression analysis to examine separate lifestyle factors related to anxiety and depression, respectively. The result of multiple linear regression was shown in Table 2. After adjusting demographic covariates, independent variables including sitting, exercise, dietary habits (regular mealtime, takeout food consumption and fruit intake), toxicant exposure, drinking and smoking were significantly associated with neither the HAM-A score nor the HAM-D score. Poor sleep quality was positively related to depressive symptoms (*P = *.018) but was not related to anxious symptoms (*P* = .188). Results in simple linear regression were consistent with those in multiple linear regression (Supplemental Digital Content 1, http://links.lww.com/MD/K550).

**Table 2:** Results of the multiple linear regression assessing lifestyle factors influencing anxiety and depression (n = 185).

Characteristic	HAM-A score		HAM-D score	
	β (SE)	*P*	β (SE)	*P*
Hematologic malignancies	0.02 (0.69)	.973	−0.24 (0.99)	.810
Age	0.09 (0.03)	.007	0.13 (0.04)	.004
Male	−0.06 (0.84)	.941	0.10 (1.21)	.931
BMI				
Underweight	3.23 (1.75)	.067	1.84 (2.50)	.464
Overweight/obese	−0.75 (0.65)	.250	−0.88 (0.93)	.346
Education				
Secondary school or less	0.09 (0.87)	.917	0.57 (1.25)	.648
University	0.56 (0.89)	.530	1.06 (1.28)	.408
Health insurance				
UEBMI	0.26 (0.88)	.767	−0.31 (1.26)	.805
URBMI	−0.10 (1.08)	.925	−1.26 (1.55)	.418
Have paid employment	−0.88 (0.92)	.340	−0.48 (1.32)	.715
High sitting time	−0.81 (0.73)	.265	−0.69 (1.04)	.504
Insufficient physical activity time	−0.18 (1.10)	.867	−0.45 (1.57)	.774
Irregular mealtime	0.23 (0.75)	.756	0.72 (1.08)	.502
Takeout food consumption in low frequency	0.90 (0.98)	.362	1.29 (1.40)	.360
Fruit intake in low frequency	0.35 (1.13)	.756	0.94 (1.62)	.564
Poor sleep quality	0.89 (0.68)	.188	2.31 (0.97)	.018
Have occupational exposure	−1.31 (1.03)	.204	−1.90 (1.47)	.199
Alcohol consumption ≥ 1 standard drink per week	0.97 (1.01)	.336	−0.22 (1.44)	.884
Smoker	−1.20 (0.89)	.180	−1.05 (1.27)	.410

BMI = body mass index, HAM-A = Hamilton Anxiety Rating Scale, HAM-D = Hamilton Depression Rating Scale, UEBMI = Urban Employee Basic Medical Insurance.

**Figure 1.**
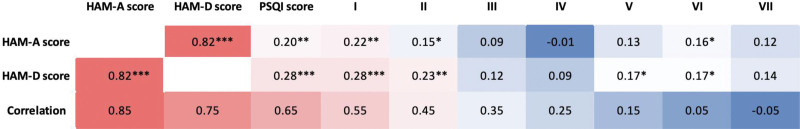
Correlation heatmap of anxiety and depression with sleep quality. I: subjective sleep quality, II: sleep latency, III: sleep duration, IV: habitual sleep efficiency, V: sleep disturbances, VI: use of sleeping medication, VII: daytime dysfunction. *P < .05, **P < .01, ***P < .001.

**Figure 2.**
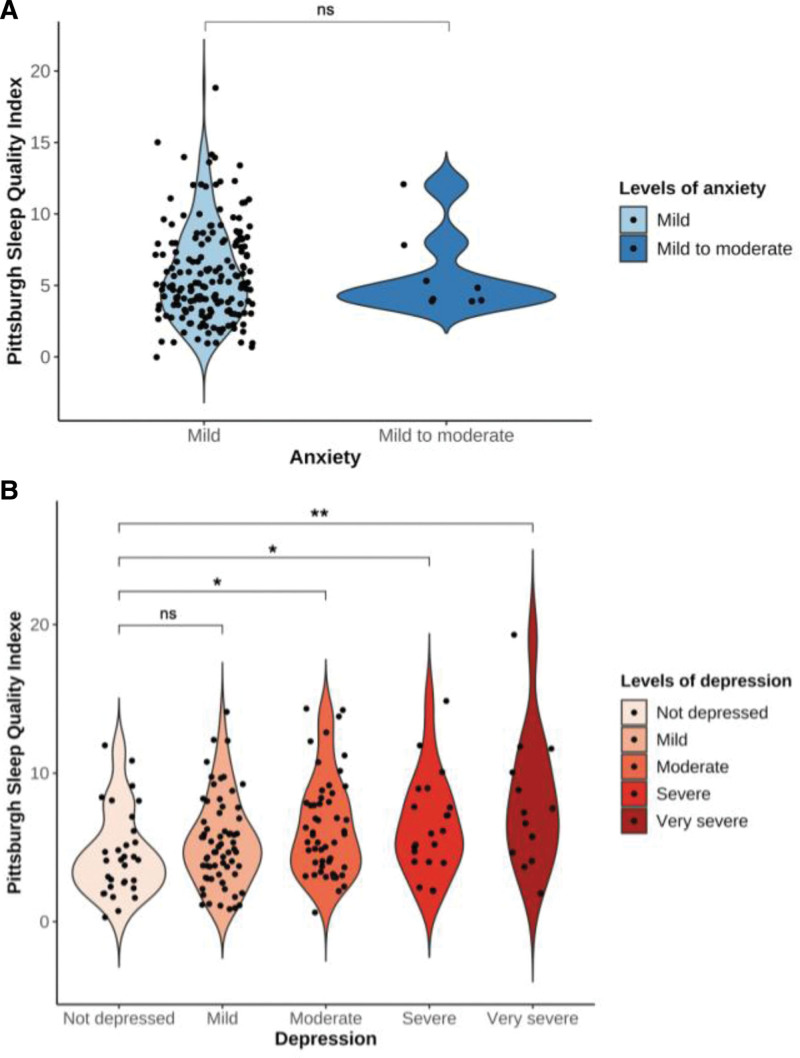
Differences in Pittsburgh Sleep Quality Index scores across different levels of anxiety (A) and depression (B). *P < .05, **P < .01. ns = not significant.

We further evaluated the correlation between sleep quality (PSQI global scores and its 7 components), anxiety and depression. Anxiety was positively correlated with depression (*r* = 0.82, *P* < .001). Subjective sleep quality, sleep latency and use of sleeping medication were positively correlated with both the HAM-A score and HAM-D score. Sleep disturbances was positively correlated with the HAM-D score (Fig. 1). The PSQI score was positively correlated with both the HAM-A score and the HAM-D score (*r* = 0.20, *P* < .01 and *r* = 0.28, *P* < .001). Additionally, we assessed PSQI scores of participants with different levels of anxiety and depression, respectively. There was no difference in PSQI scores between mild and mild to moderate anxiety (Fig. 2A). However, we observed the increased PSQI score was related to more serious depressive symptoms (Fig. 2B).

### 3.4. Association of clustering of lifestyle behaviors with anxiety and depression

**Figure 3.**
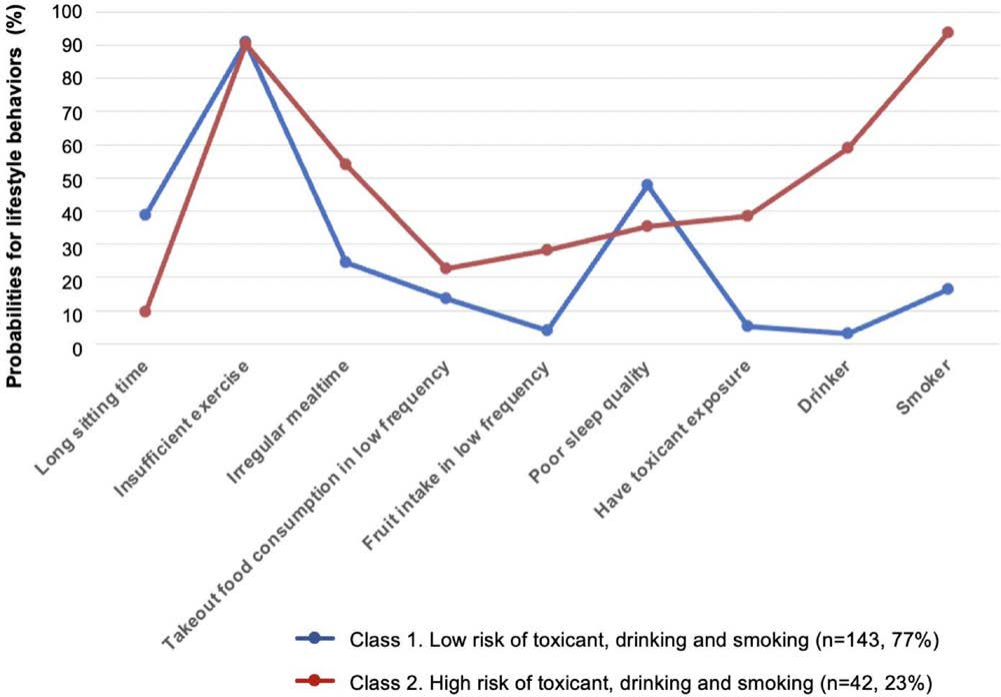
Two-class lifestyle patterns were identified by latent class analysis. Shown were item-response probabilities (y-axis) for lifestyle behaviors (x-axis).

The 2 classes were selected considering a lower adjusted Akaike information criterion and a lower adjusted Bayesian information criterion (Table 3). The 2 classes were named to best represent the characteristics of patients’ lifestyle according to the response probabilities for 6 indicators, including mealtime, takeout food consumption, fruit intake, toxicant exposure, drinking and smoking (Fig. 3). Class 1 accounted for 77% of the participants and was characterized by a “Low risk of dietary habits, toxicant, drinking and smoking” profile. In contrary, Class 2 (23%, n = 42) presented a greater likelihood of having poor dietary habits, toxicant exposure, drinking and smoking, and thus was characterized by a “High risk of dietary habits, toxicant, drinking and smoking” profile. However, patients in Class 2 had lower probabilities of long sitting time and poor sleep quality, and they had a similar likelihood of exercise time compared with those in Class 1.

**Table 3:** Fit statistics of latent classes of lifestyle behaviors.

#Latent classes	G^2^	Degree of freedom	AIC	Consistent AIC	BIC	Adjusted BIC	Entropy
1	261.97	176	1671.92	1709.91	1700.91	1672.40	–
2	174.88	166	1604.83	1685.02	1666.02	1605.84	0.73
3	156.38	156	1606.34	1728.73	1699.73	1607.88	0.66
4	137.28	146	1607.23	1771.82	1732.82	1609.30	0.87
5	117.34	136	1607.29	1814.09	1765.09	1609.89	0.90

AIC = Akaike information criterion, BIC = Bayesian information criterion.

We further used logistic regression to investigate the association of lifestyle classes with anxiety and depression, respectively. The Sociodemographic and mental health measures were included as independent variables to predict the class membership (Table 4). Majority of subjects in Class 2 were male and had a lower level of education. However, there were no statistical differences of HAM-A scores or HAM-D scores between the 2 class memberships. The distribution of HAM-A scores and HAM-D scores among patients classified by class membership was displayed in Supplemental Digital Content 2, http://links.lww.com/MD/K551.

**Table 4:** Odds of latent class membership by sociodemographic and mental health measures.

Variables	Class2 (n = 42)	
	OR (95% CI)	*P*
Age	1.00 (0.97, 1.04)	.922
Male	24.97 (5.32, 117.11)	<.001
BMI		
Underweight	0.52 (0.04, 7.03)	.620
Overweight/obese	1.21 (0.52, 2.84)	.657
Education		
Secondary school or less	3.71 (1.21, 11.38)	.022
University	0.87 (0.25, 2.99)	.818
Health insurance		
UEBMI	1.26 (0.39, 4.09)	.695
URBMI	1.17 (0.27, 5.03)	.835
Have paid employment	3.13 (0.55, 17.73)	.195
Hematologic neoplasms	0.96 (0.36, 2.55)	.940
HAM-A score	0.97 (0.82, 1.16)	.771
HAM-D score	1.02 (0.91, 1.15)	.706

The reference group = Class1: Low risk of toxicant, drinking and smoking (n = 143).

BMI = body mass index, CI = confidence interval, HAM-A = Hamilton Anxiety Rating Scale, HAM-D = Hamilton Depression Rating Scale, OR = odds ratio, UEBMI = Urban Employee Basic Medical Insurance.

## 4. Discussion

The study focused on the association of both separate and combined lifestyle behaviors with anxiety and depression in individuals with hematologic disorders using regression analysis and latent class analysis. Sitting and exercise, dietary habits, toxicant exposure, drinking and smoking had no association with anxiety and depression. The global score on the PSQI was positively correlated with the scores for both anxiety and depression. Further research on latent classes of lifestyle behaviors found that there was no association between class memberships and symptoms of anxiety and depression. These findings should be taken into consideration while formulating health promotion strategies for patients with hematologic disorders.

In our study, many separate lifestyle behaviors had no association with anxiety and depression. The possible explanation was the nature of hematologic disorders and the potential side effects of treatment, had a greater impact on anxiety and depression than health behaviors.^[38,39]^ Hematologic diseases can cause symptoms such as fatigue, pain and difficulty breathing, which can lower a patient’s quality of life and exacerbate negative feelings. Additionally, the uncertainty and unpredictability of the disease course, as well as financial stress, can further increase their phycological and psychological burden.^[40]^ However, it is worth noting that practicing healthy behaviors, such as balanced and healthy eating, can benefit individuals with hematologic disorders, including improving overall health and preventing the development of other hematologic disorders, such as megaloblastic anemia and iron deficiency anemia. Sleep plays a vital role in regulating our emotions. Lack of sleep can lead individuals to be more irritable, easily frustrated, and less able to cope with stressors. Chronic sleep deprivation can also interfere with the production of serotonin and other neurotransmitters that regulate mood, leading to a greater risk of developing anxiety and depression.^[41,42]^ It is essential to prioritize good sleep hygiene, such as maintaining a consistent sleep schedule to strength body’s sleep-wake cycle. The recommended amount of sleep is at least 7 hours per night.^[43,44]^

Clustering of lifestyles were independent of anxiety and depression, which were inconsistent with those of previous studies.^[45–47]^ This difference may be attributed to the following. First, lifestyle modifications may not have a similar impact on mental issues in all populations. Compared with individuals in those studies including healthy subjects or patients at risk of cardiovascular disease, hematological patients may experience higher levels of anxiety and depression due to the feature of the illness. Second, variations in the measurement of lifestyle behaviors and mental health outcomes may contribute to the observed discrepancy. For instance, the health behaviors in Bonnet’s study^[46]^ were measured quantitatively, whereas our study used qualitative measures for lifestyles. Third, the sample size of our study being limited may have made it challenging to draw statistically significant conclusions.^[48]^

It was the first study to report the association of both separate and combined lifestyle behaviors with anxiety and depression among hematological patients using a more comprehensive lifestyle survey. The clustering in this study utilized a latent class model, which is considered more sophisticated than traditional clustering methods as it does not rely on arbitrary cluster criteria. Nevertheless, there were several limitations. First, the lifestyle behaviors and mental measures were assessed at a certain time-point due to the cross-sectional study design. Hence, they can only provide a snapshot of the study population, which are unable to identify the causal relationship between health behaviors and mental health. Second, all information on lifestyles was self-reported and thus potentially lead to information bias. Furthermore, the study population was individuals who positively received the treatment and the sample size was limited. The generalization of the obtained results should be a concern.

## 5. Conclusion

In this study, we found good sleep quality was inversely correlated with anxiety and depression. However, other lifestyles were independent of anxious and depressive symptoms among patients with hematologic disorders. Moreover, lifestyle behaviors of identified 2 classes did not commonly affect the mental condition. Our findings suggested that healthcare providers should prioritize the evaluation of sleep quality during hospitalization and manage sleep problems to improve the mental health of hematological patients.

## Author contributions

**Conceptualization:** Baoxin Shi.

**Data curation:** Jing Xu, Zhiqiong Wei, Liyun Li, Jin Dong, Xiao Yu, Juan Li, Juanjuan Zhao.

**Formal analysis:** Xia Li, Jingyu Zhao.

**Investigation:** Zhexiang Kuang, Bin Zhang, Baoxin Shi.

**Methodology:** Zhexiang Kuang.

**Project administration:** Zhexiang Kuang, Baoxin Shi.

**Supervision:** Bin Zhang, Xia Li, Baoxin Shi.

**Writing – original draft:** Zhexiang Kuang, Jingyu Zhao.

**Writing – review & editing:** Bin Zhang, Xia Li, Baoxin Shi.

## Supplementary Material




