# Supplementary material for: Evaluation of lifestyle behaviors, anxiety and depression in patients with hematologic disorders
Source: Medicine (Baltimore). 2023 Nov 17;102(46):e35863. doi: 10.1097/MD.0000000000035863 (PMC10659686; doi:10.1097/MD.0000000000035863)
Supplement: Supplementary file 1 [file medi-102-e35863-s001.docx]

**Table S1** Results of the simple linear regression assessing lifestyle factors influencing anxiety and depression with covariates (n=185)

| **Characteristic** | **HAM-A score** | |  | **HAM-D score** | |
| --- | --- | --- | --- | --- | --- |
|  | $\boldsymbol{\beta}$**(*SE*)** | ***p*** |  | $\boldsymbol{\beta}$**(*SE*)** | ***p*** |
| Long sitting time | -0.86 (0.69) | 0.21 |  | -1.12 (0.99) | 0.26 |
| Insufficient exercise | -0.14 (1.08) | 0.90 |  | -0.19 (1.55) | 0.90 |
| Irregular mealtime | 0.03 (0.71) | 0.97 |  | 0.26 (1.02) | 0.80 |
| Takeout food consumption in low frequency | -0.17 (0.88) | 0.85 |  | -0.21 (1.27) | 0.87 |
| Fruit intake in low frequency | 0.78 (1.12) | 0.49 |  | 1.23 (1.61) | 0.45 |
| Poor sleep quality | 0.96 (0.63) | 0.13 |  | 2.72 (0.90) | 0.003 |
| Have toxicant exposure | -1.09 (1.01) | 0.28 |  | -1.23 (1.45) | 0.40 |
| Drinking ≥ 1 standard drink per week | 0.65 (0.97) | 0.50 |  | -0.06 (1.39) | 0.97 |
| Smoker | -1.17 (0.77) | 0.13 |  | -0.78 (1.11) | 0.48 |
