# Supplementary material for: Evaluation of lifestyle behaviors, anxiety and depression in patients with hematologic disorders
Source: Medicine (Baltimore). 2023 Nov 17;102(46):e35863. doi: 10.1097/MD.0000000000035863 (PMC10659686; doi:10.1097/MD.0000000000035863)
Supplement: Supplementary file 2 [file medi-102-e35863-s002.docx]

1. (b)


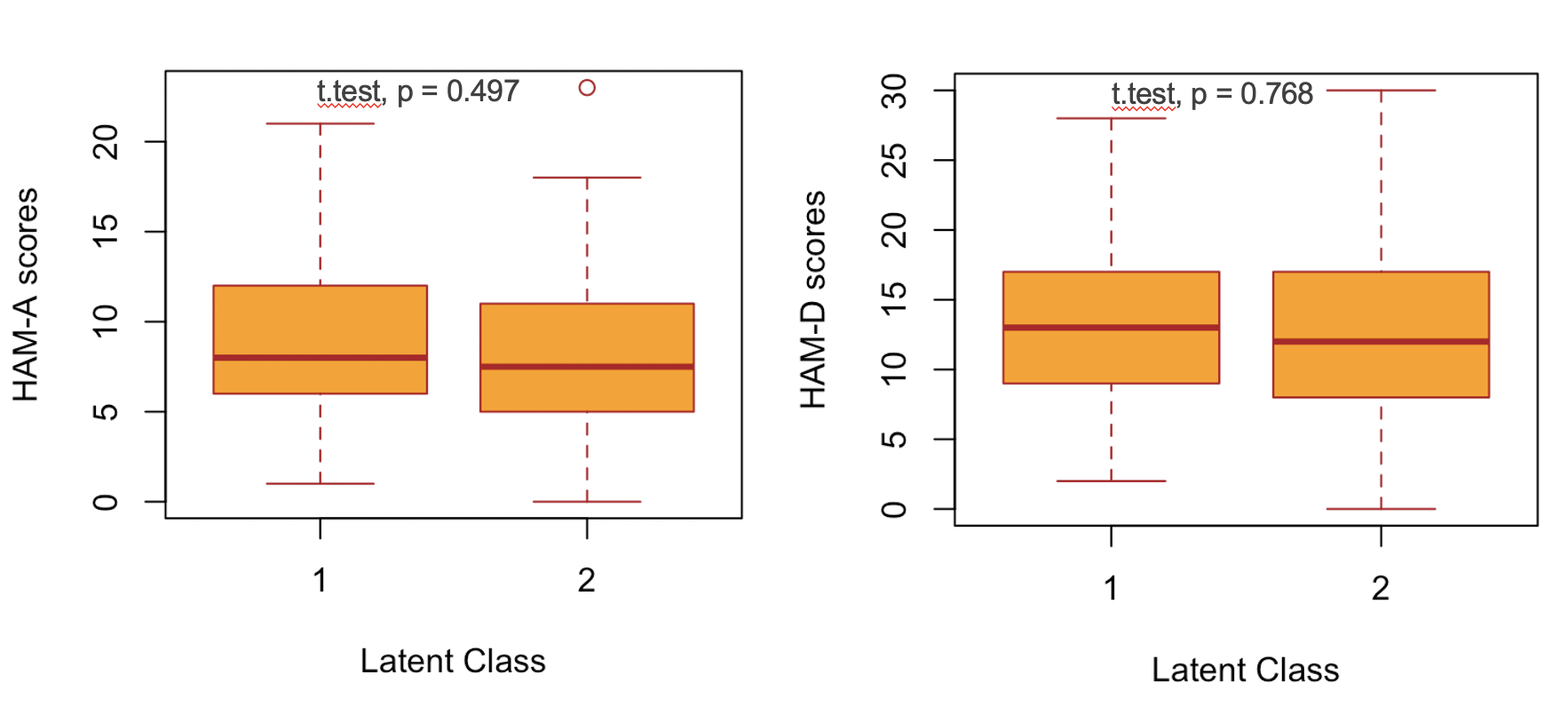


**Figure S1** Differences in HAM-A scores(a) and HAM-D scores(b) between the two class memberships (a) anxiety.
